# Comparison of Treatment Modalities for Dry Eye in Primary Sjögren’s Syndrome

**DOI:** 10.3390/jcm11020463

**Published:** 2022-01-17

**Authors:** Hyunmin Ahn, Yong Woo Ji, Ikhyun Jun, Tae-im Kim, Hyung Keun Lee, Kyoung Yul Seo

**Affiliations:** 1 Institute of Vision Research, Department of Ophthalmology, Yonsei University College of Medicine, Seoul 03722, Korea; overhyun31@gmail.com (H.A.); lusita30@yuhs.ac (Y.W.J.); hadesdual@yuhs.ac (I.J.); tikim@yuhs.ac (T.-i.K.); shadik@yuhs.ac (H.K.L.); 2 Corneal Dystrophy Research Institute, Yonsei University College of Medicine, Seoul 03722, Korea

**Keywords:** dry eye, ocular surface inflammation, punctal plug, Sjögren’s syndrome

## Abstract

Purpose: To evaluate the effectiveness of different treatment modalities for dry eye in primary Sjögren’s syndrome with their potential overlapping influences. Methods: This study included 199 patients with newly diagnosed primary Sjögren’s syndrome from 2005 to 2020. Various treatment modalities for primary Sjögren’s syndrome were compared. Improvement of corneal staining based on Sjögren’s International Collaborative Clinical Alliance (SICCA) scores was the primary outcome. Results: The average follow-up period was 5.4 ± 3.1 (range, 2.0–14.1) years. Analysis of the individual treatments showed that punctal plug insertions in the lower and upper eyelids were strongly associated with improvement of SICCA scores (β = 2.70 and 1.80, *p* < 0.001 and <0.001, respectively). With ocular surface inflammation, corneal staining scores improved significantly with steroid eye drops. Prednisolone (1%) had the strongest association with improvement of corneal staining scores (β = 1.48, *p* < 0.001); this was based on the frequency of administration. Without ocular surface inflammation, diquafosol (3%), carbomer gel, and lanolin ointment were effective (β = 1.37, 1.06, and 1.17; *p* = 0.003, 0.003, and <0.001, respectively). Conclusions: Punctal plug insertion, primarily targeting aqueous deficiency, is the mainstay of the treatment for dry eye in primary Sjögren’s syndrome even in the presence of ocular surface inflammation. Furthermore, the effectiveness of treatment modalities for dry eye in primary Sjögren’s syndrome was dependent on the presence of ocular surface inflammation.

## 1. Introduction

Sjögren’s syndrome (SS) is a long-term, progressive autoimmune disease that affects the exocrine glands, including the lacrimal and salivary glands. Its symptoms include dryness of the eyes and mouth, pain, and fatigue [1]. SS is one of the most common autoimmune diseases. Its phenotypic expression varies with geolocation and ethnicity. SS affects approximately 4,000,000 Americans. Its highest incidence (43 per 100,000 people) is registered in Europe and Asia [1,2,3].

Regarding symptoms and signs of dry eye disease, patients with SS have a more severe clinical presentation than those with non-SS. Furthermore, not only are the level of tear inflammatory cytokines elevated, but the composition of inflammatory cytokines are different from those with non-SS [4]. While this makes the treatment of SS challenging, there are various available treatment modalities. Several studies have explored the treatment of dry eye in primary SS [5,6]; however, these are limited owing to the small sample sizes and short follow-up periods. Clinical guidelines for the management of dry eye disease in SS are based on the effects of individual treatments [7]. However, in clinical situations, SS is rarely treated using a single treatment agent.

No studies have compared and analyzed the effects of simultaneous, long-term, multiple treatments. Since the treatment mechanisms in dry eye are not mutually exclusive but rather interconnected [8], it is necessary to determine the effectiveness of the individual treatments with their potential overlapping influences. The purpose of this study was to evaluate the comparative effects of treatments administered concurrently for primary SS using long-term clinical data and to suggest the therapeutic edge for the treatment of primary SS in the real world.

## 2. Methods

This observational study was conducted at Severance Hospital, Yonsei University, College of Medicine, South Korea, between January 2005 and December 2020. The Severance Hospital Clinical Research Ethics Committee approved the study protocol (IRB protocol number-4-2021-0682). The study was conducted in accordance with the tenets of the Declaration of Helsinki.

### 2.1. Participants

All new patients with primary SS who were followed up for at least 2 years were enrolled in the study. Diagnosis of primary SS was based on the American-European Consensus Group 2002 definition and the revised 2016 criteria, including findings of systemic, serological, and immunological examinations [9,10]. Patients with concomitant autoimmune diseases or secondary SS were excluded. Patients with other ocular surface diseases that were not related to SS were excluded.

### 2.2. Clinical Assessments

The therapeutic effect of the treatments for primary SS were evaluated based on two clinical findings: corneal staining scores (CSS) with fluorescein and dry eye symptoms based on the quantitative ocular grading system in the Sjögren’s International Collaborative Clinical Alliance (SICCA) registry [11,12]. Binary assessments evaluating the improvement in clinical signs and symptoms were conducted. An improvement of the CSS, shown by an improvement of the CSS grade (grade 0 (0 dots), grade 1 (1–5 dots), grade 2 (6–30 dots), and grade 3 (over 30 dots)) or CSS grade 0, was considered the primary endpoint. Symptoms were evaluated on a 5-point scale measuring the extent of the influence of ocular surface discomfort on the functional impairment and quality of daily, social, or occupational life affected by ocular surface discomfort (very good (no symptoms and no concern), good (minimal symptoms but no concern), satisfactory (acceptable symptoms), poor (symptoms affecting quality of life), and very poor (symptoms that severely impair quality of life)). Improvement in the symptoms (seen as improvement in the symptom grade) and limited effect of dry eye symptoms on daily life (better than “good”) were considered as secondary endpoints. Patients with an Efron conjunctival hyperemia scale score ≥2 were considered as having ocular surface inflammation [13,14].

All patients received a combined therapy with artificial tears, and the therapy was modulated step-by-step. Individual treatment plans were established when treatment plans were changed based on the methods or frequencies of the medications or procedures. The treatment methods and frequencies were set as independent variables in each treatment plan.

### 2.3. Statistical Analysis

A mixed-effects logistic regression model was used to compare the treatments for the primary and secondary outcomes. The statistical model was selected to control for various treatments, observation interval, and intra-subject variation. The individual treatments, baseline CSS, and presence of ocular surface inflammation were considered fixed effects, and patient factors were considered as random effects. A mixed-effects regression model must assume normal distribution and homogeneity of variance; therefore, the treatments that violated this statistical assumption were excluded from the analysis. Finally, 11 treatments (oral pilocarpine; low-dose oral steroid; tear substitutes (carbomer gel, lanoline ointment, and 3% diquafosol); topical cyclosporine; topical steroid with 1% prednisolone, 0.5% loteprednol, and 0.1% fluorometholone;^7^ and long-term punctal plug insertion (upper and lower)) were included.

The model was analyzed in two steps. First, the variables were divided into binary or qualitative categories as shown in Table 1, and the significant parameters were then subjected to the second step. Second, the variables were converted into quantitative values as dose or frequency as shown in Supplementary materials Table S1. A stratified analysis for ocular surface inflammation was performed. *p*-Values of <0.05 were considered statistically significant. All estimated coefficients (exp(B) or β) and 95% confidence intervals (CI) were calculated.

## 3. Results

In total, 256 patients were screened. Eighteen patients were excluded owing to secondary or concomitant autoimmune diseases, rheumatoid arthritis, systemic lupus erythematosus, or IgG4-related disease. Patients with glaucoma (*n* = 5), patients with a follow-up period of less than two years (*n* = 5), patients who were enrolled in other studies (*n* = 3), and patients with disorders that affected eyelid function (*n* = 2; facial palsy and hemifacial spasm) were also excluded. Patients whose baseline CSS was grade 0 or whose baseline symptom degree was better than “good” were excluded (*n* = 24). One hundred and twenty-four treatment plans (3.45% of total treatment plans) were excluded based on the assumptions of the mixed-effects model. Finally, 199 patients with primary SS and 3468 treatment plans were evaluated.

The baseline demographics and characteristics are shown in Table 1. The average observation period was 5.4 ± 3.1 years (range, 2.0–14.1 years), 95% of participants were female, and the mean age was 52.3 ± 12.2 years. Throughout the entire observational period, 17.8% of patients (29.1% of treatment plans) were in the ocular surface inflammatory phase. For the baseline CSS, 20.1% of the eyes scored grade 1, 46.0% grade 2, and 33.9% grade 3. CSS and ocular surface inflammation significantly affected the primary outcome (*p* < 0.001 and 0.005, respectively). The higher the CSS, the lower the improvement of CSS (grade 1 vs. grade 2, β = 0.61 (95% CI, 0.49 to 0.76), *p* < 0.001; grade 1 vs. grade 3, β = 0.51 (95% CI, 0.39 to 0.66), *p* < 0.001). CSS did not improve as much when ocular surface inflammation was present (β = 0.88 (95% CI, 0.84 to 0.92)).

**Figure 1:**
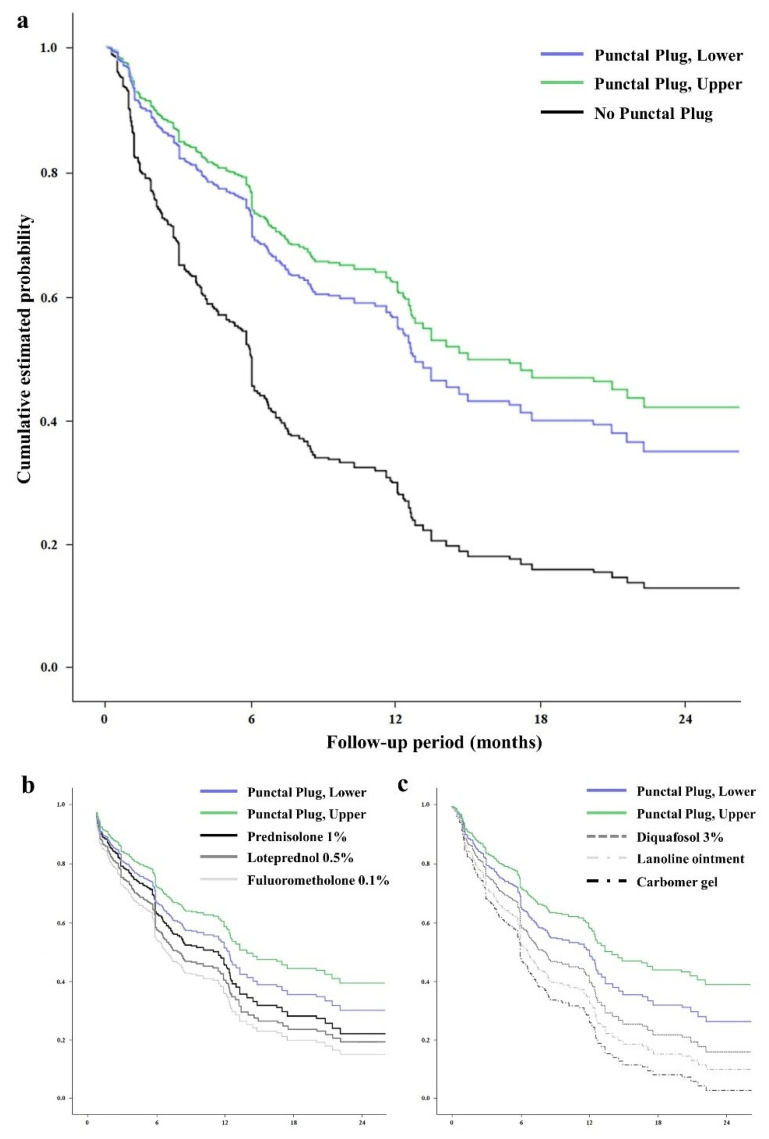
The (relative) cumulative estimated probability of the individual treatments for on corneal staining score in primary Sjögren’s syndrome. The graphs represent the relative value according to the follow-up to the initial value. The effects of punctal plug insertion for both the lower and upper eyelid in overall data (**a**). The effects of the individual treatments for on the disease improvement with (**b**) or without (**c**) ocular surface inflammation.

The therapeutic effects of the individual treatments for improvement of CSS, following adjustment for baseline CSS and ocular surface inflammation, are presented in Table 2 and Figure 1a. Punctal plug insertions in the lower and upper eyelids were significantly effective (*p* < 0.001 and <0.001, respectively). Carbomer gel, lanoline ointment, diquafosol, and steroid eye drops (1% prednisolone, 0.5% loteprednol, and 0.1% fluorometholone) were significantly effective (*p* = 0.037, 0.033, 0.005, 0.009, 0.042, and 0.035, respectively).

The therapeutic effects of individual treatments for symptom improvement are presented in Table 3. Punctal plug insertion in the lower and upper eyelids improved symptoms significantly (*p* < 0.001 and <0.001, respectively). Lanoline lubricating ointment, 3% diquafosol, and all steroid eye drops (1% prednisolone, 0.5% loteprednol, and 0.1% fluorometholone) were significantly effective (*p* = 0.023, 0.022, <0.001, 0.017, and <0.001, respectively).

With ocular surface inflammation (Table 4 and Figure 1b), punctal plug insertions in the lower and upper eyelids were significantly effective (ß = 2.26 and 1.70, *p* < 0.001 and 0.002, respectively). Steroid eye drops and punctal plug insertions were effective. The effects of the steroid eye drops depended on the frequency of daily use, ranging from 1 to 4 times per day. Prednisolone (1%) was the most effective steroid eye drop (β = 1.48, *p* < 0.001), followed by 0.5% loteprednol (β = 1.39, *p* = 0.012) and 0.1% fluorometholone (β = 1.25, *p* < 0.001).

Without ocular surface inflammation (Table 5 and Figure 1c), punctal plug insertions in the lower and upper eyelids were significantly effective (β = 2.97 and 1.85, *p* < 0.001 and <0.001, respectively). Carbomer gel, lanoline ointment, and 3% diquafosol were significantly effective (β = 1.06, 1.17, and 1.37; *p* = 0.003, <0.001, and 0.003, respectively).

**Figure 2:**
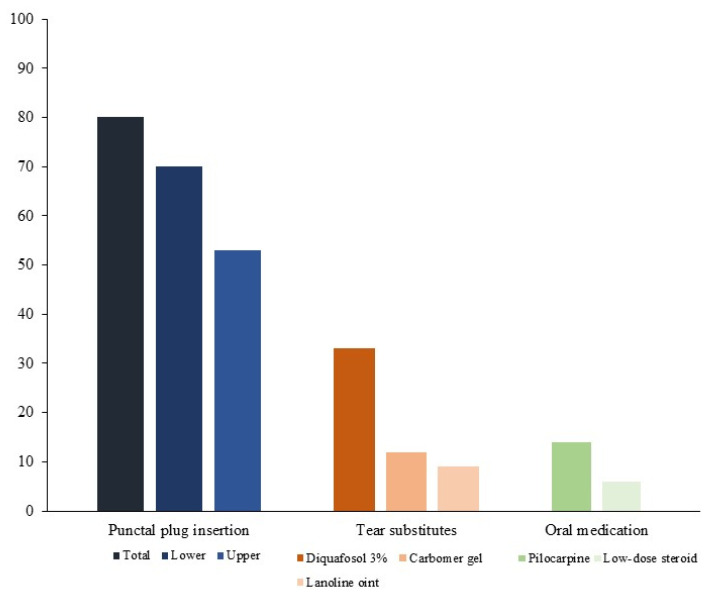
The proportions of the maintenance treatments for non-inflammatory period of primary Sjögren syndrome.

To show the long-term treatment modalities, we evaluated the proportions of maintenance treatments used without disease deterioration (Figure 2). Long-term punctal plug insertion was used in 80% of patients. Oral pilocarpine was used in 14% and oral low-dose steroid in 6% of the patients. Gel- and ointment-forming tear substitutes were used in 21%. Diquafosol (3%) was used in 33% of the patients.

## 4. Discussion

Previous studies determined that various treatment modalities were effective for SS [5,6], but the results of the studies were focused on single treatment agents. Multiple treatments were administered in no study, and the treatment modality to be prioritized in actual clinical practice is still unclear. This study involved multiple treatment modalities with simultaneous interactions; the effectiveness of punctal plug insertions, especially in the inferior lacrimal punctum, which play a significant role in tear drainage and result in retention of the aqueous component of the tear film [15], was the most prominent. SS is an autoimmune disease that causes dry eyes due to lack of the aqueous component of the tear film. Our study showed that treatment for aqueous deficiency, which is the key etiology of the disease, is more important than other treatment modalities and that tear retention can reduce the frictional force that caused mechanical trauma and consequent inflammation [8,16]. 

Multiple studies have shown the clinical effect of topical steroids on CSS [17,18]. However, no study clearly analyzed the effect according to potency and frequency of steroid eye drops. In our study, 1% prednisolone, 0.5% loteprednol, and 0.1% fluorometholone were effective, but this depended on the frequency of administration and presence of ocular surface inflammation. Additionally, we observed that in primary SS, the higher the potency of the steroid medication, the higher the treatment efficacy [19,20]. This result confirmed once again that primary SS is an inflammatory disease and showed that a treatment modality that targets inflammation, depending on its potency and frequency, is important to control the disease.

Among the tear substitutes, 3% diquafosol was superior to the other tear substitutes in patients without inflammation although gels and ointments are known to help with water, lipid, and mucin retention [21,22]. Diquafosol, an agonist of the P2Y_2_ purinergic receptor, promotes mucin secretion and membrane-associated mucin expression on the ocular surface [23,24,25], thus maintaining the aqueous component of the tear film and improving dry eye symptoms and signs [26].

Our study was designed to analyze the comparative effects of treatments for primary SS and does not reflect the absolute effect of individual treatments. This could be a limitation to our study. Modulating or interpreting statistical modeling outputs is difficult in a multivariate analysis [27]. For the modeling process, the dependent variables were simplified to a binary classification. A step-by-step approach for the independent, qualitative, and quantitative variables was used. However, the aim of this study was to identify which treatment should be prioritized and to know whether inflammation significantly affects treatment efficacy in clinical practice; the simplified results provide a clear clinical approach.

## 5. Conclusions

Punctal plug insertion, which primarily targets aqueous deficiency, is the mainstay of the treatment for dry eyes in primary SS, even in the presence of ocular surface inflammation. Punctal plug insertion can be recommended for use in combined treatments for dry eyes in primary SS. Because the long-term effectiveness of treatments for dry eyes in primary SS was dependent on the presence of ocular surface inflammation, short-term use of steroid eye drops can be considered in the presence of inflammation, and a topical mucin secretagogue can be helpful in the absence of inflammation.

## Figures and Tables

**Table 1:** The baseline demographics and the study characteristics.

Characteristic	(*n* = 191)
Age (years, mean ± SD)	52.3 ± 12.2 (range 35 to 90)
Sex (Female, %)	95
Observation period (years, mean ±SD)	5.4 ± 3.1 (range 2.0 to 14.1)
Ocular Surface Inflammation period (%)	17.8, of entire observational period (29.1, of treatment plans)
Corneal Staining Score * (%)	
Grade 1	20.1
Grade 2	46.0
Grade 3	33.9
The Symptom Scale (%)	
Satisfactory	18.5
Poor	53.8
Very poor	27.7

* based on the quantitative ocular grading system by the Sjögren’s International Collaborative Clinical Alliance (SICCA).

**Table 2:** Therapeutic effects of the treatments for improvement of corneal staining score (SICCA).

		95% CI		
Treatments	ß	Lower	Upper	*p*
Oral pilocarpine	1.017	0.995	1.039	0.141
Low-dose oral steroid	1.220	0.724	2.057	0.455
Tear substitutes				
Carbomer gel	1.035	1.002	1.069	0.037 *
Lanoline ointment	1.142	1.008	1.274	0.033 *
Diquafosol 3%	1.203	1.060	1.475	0.005 *
Topical cyclosporine	1.082	0.980	1.191	0.099
Steroid eye drops (frequency dependent)				
Prednisolone 1%	1.255	1.058	1.487	0.009 *
Loteprednol 0.5%	1.229	1.002	1.486	0.042 *
Fluorometholone 0.1%	1.188	1.012	1.395	0.035 *
Punctal plug insertion				
Lower eyelid	2.697	2.161	3.365	<0.001 *
Upper eyelid	1.801	1.369	2.370	<0.001 *

Adjustment for corneal staining score and ocular surface inflammation. * *p* < 0.05.

**Table 3:** Therapeutic effects of the treatments for improvement of symptoms.

		95% CI		
Treatments	ß	Lower	Upper	*p*
Oral pilocarpine	0.987	0.962	1.013	0.987
Low-dose oral steroid	0.972	0.882	1.070	0.972
Tear substitutes				
Carbomer gel	1.030	0.996	1.065	0.089
Lanoline ointment	1.040	1.006	1.077	0.023 *
Diquafosol 3%	1.027	1.003	1.052	0.022 *
Topical cyclosporine	0.920	0.836	1.013	0.136
Steroid eye drops (frequency dependent)				
Prednisolone 1%	1.297	1.174	1.434	<0.001 *
Loteprednol 0.5%	1.228	1.034	1.542	0.017 *
Fluorometholone 0.1%	1.228	1.124	1.343	<0.001 *
Punctal plug insertion				
Lower eyelid	2.867	2.248	3.655	<0.001 *
Upper eyelid	2.102	1.535	2.877	<0.001 *

Adjustment for corneal staining score and ocular surface inflammation. * *p* < 0.05.

**Table 4:** Therapeutic effects of the treatments for improvement of primary Sjögren syndrome with ocular surface inflammation.

		95% CI		
Treatments	ß	Lower	Upper	*p*
Oral pilocarpine	1.050	1.000	1.102	0.092
Low-dose oral steroid	1.250	0.875	1.745	0.278
Tear substitutes				
Carbomer gel	0.986	0.888	1.094	0.786
Lanoline ointment	1.097	0.948	1.269	0.214
Diquafosol 3%	1.012	0.924	1.108	0.800
Topical cyclosporine	1.084	0.980	1.199	0.098
Steroid eye drops (frequency dependent)				
Prednisolone 1%	1.477	1.292	1.688	<0.001 *
Loteprednol 0.5%	1.390	1.076	1.798	0.012 *
Fluorometholone 0.1%	1.245	1.149	1.350	<0.001 *
Punctal plug insertion				
Lower eyelid	2.257	1.927	2.643	<0.001 *
Upper eyelid	1.702	1.402	2.067	0.002 *

Adjustment for corneal staining score. * *p* < 0.05.

**Table 5:** Therapeutic effects of the treatments for improvement of primary Sjögren syndrome without ocular surface inflammation.

		95% CI		
Treatments	ß	Lower	Upper	*p*
Oral pilocarpine	0.991	0.962	1.022	0.578
Low-dose oral steroid	0.992	0.901	1.091	0.862
Tear substitutes				
Carbomer gel	1.060	1.019	1.102	0.003 *
Lanoline ointment	1.171	1.134	1.211	<0.001 *
Diquafosol 3%	1.372	1.116	1.685	0.003 *
Topical cyclosporine	0.861	0.686	1.080	0.196
Punctal plug insertion				
Lower eyelid	2.967	2.259	3.897	<0.001 *
Upper eyelid	1.848	1.315	2.598	<0.001 *

Adjustment for corneal staining score. * *p* < 0.05.

## Supplementary Materials

The following supporting information can be downloaded at: https://www.mdpi.com/article/10.3390/jcm11020463/s1, Table S1: The independent variables of treatment modalities in this study.
