# Supplementary material for: Comparison of Treatment Modalities for Dry Eye in Primary Sjögren’s Syndrome
Source: J Clin Med. 2022 Jan 17;11(2):463. doi: 10.3390/jcm11020463 (PMC8781254; doi:10.3390/jcm11020463)
Supplement: Supplementary file 1 [file jcm-11-00463-s001.zip › jcm-1545920-supplementary.pdf]

Supplementary Table S1. The independent variables of treatment modalities in this study.

Treatments	Qualitative values	Quantitative values
Oral Pilocarpine	2.5 mg to 20 mg daily / not	daily dose
Low-dose oral prednisolone (or alternative steroid)	2.5 mg to 10 mg daily / not	daily dose
Tear substitutes		
Carbomer	1-24 times daily / not	daily frequency
Lanoline	1-24 times daily / not	daily frequency
Diquafosol 3%	5-12 times daily / not	daily frequency
Topical cyclosporine 0.05%	2-4 times daily / not	daily frequency
Pulsed steroid eye drops		
Prednisolone 1%	0.5-4 times daily / not	daily frequency
Loteprednol 0.5%	0.5-4 times daily / not	daily frequency
Fluorometholone 0.1%	1-4 times daily / not	daily frequency
Punctal plug insertion		
Lower eyelid	Used / not	-
Upper eyelid	Used / not	-
